# Recent Studies in Photodynamic Therapy for Cancer Treatment: From Basic Research to Clinical Trials

**DOI:** 10.3390/pharmaceutics15092257

**Published:** 2023-08-31

**Authors:** Tae Eun Kim, Ji-Eun Chang

**Affiliations:** College of Pharmacy, Dongduk Women’s University, Seoul 02748, Republic of Korea

**Keywords:** photodynamic therapy, cancer treatment, photosensitizers, lung cancer, head and neck cancer, non-melanoma skin cancer, prostate cancer, breast cancer

## Abstract

Photodynamic therapy (PDT) is an emerging and less invasive treatment modality for various types of cancer. This review provides an overview of recent trends in PDT research, ranging from basic research to ongoing clinical trials, focusing on different cancer types. Lung cancer, head and neck cancer, non-melanoma skin cancer, prostate cancer, and breast cancer are discussed in this context. In lung cancer, porfimer sodium, chlorin e6, and verteporfin have shown promising results in preclinical studies and clinical trials. For head and neck cancer, PDT has demonstrated effectiveness as an adjuvant treatment after surgery. PDT with temoporfin, redaporfin, photochlor, and IR700 shows potential in early stage larynx cancer and recurrent head and neck carcinoma. Non-melanoma skin cancer has been effectively treated with PDT using methyl aminolevulinate and 5-aminolevulinic acid. In prostate cancer and breast cancer, PDT research is focused on developing targeted photosensitizers to improve tumor-specific uptake and treatment response. In conclusion, PDT continues to evolve as a promising cancer treatment strategy, with ongoing research spanning from fundamental investigations to clinical trials, exploring various photosensitizers and treatment combinations. This review sheds light on the recent advancements in PDT for cancer therapy and highlights its potential for personalized and targeted treatments.

## 1. Introduction

Photodynamic therapy (PDT) has garnered attention as a less invasive treatment option for various cancer types and infectious diseases, including bacteria-infected wounds, acne vulgaris, human papillomavirus viral warts, and cutaneous leishmaniasis [1,2,3]. Notably, PDT demonstrates remarkable advantages in treating solid tumors.

Despite advancements in cancer research and treatment, cancer remains a leading cause of death worldwide. According to GLOBOCAN 2020, there were 19.3 million newly diagnosed cancer cases and 10.0 million cancer-related deaths. Projections indicate that the number of cancer cases may rise to 28.4 million by 2040 [4]. Consequently, the development of novel and effective cancer therapies is of utmost importance.

Conventional cancer treatments such as surgery, chemotherapy, and radiation therapy have been the primary approaches [5,6]. While surgery is typically preferred for early stage solid tumors, several factors, including tumor burden, location, type, and performance status, may limit the feasibility of surgical resection. Moreover, surgical interventions often necessitate additional adjuvant therapies like chemotherapy and radiation, both of which can lead to various adverse effects, including hair loss, weight loss, fatigue, nausea, and vomiting.

PDT has emerged as a promising alternative cancer therapy due to its potential for high cancer selectivity, potentially leading to increased efficacy at the tumor site with reduced toxicity to normal tissue [7]. PDT relies on three essential components: photosensitizers, a specific wavelength of light, and an adequate supply of molecular oxygen [8,9,10,11]. Photosensitizers tend to accumulate more in tumor tissues compared to normal tissues, and although the exact mechanisms for this selective tumor localization are not fully understood, characteristics unique to tumors, such as leaky vasculature, decreased pH, overexpressed low-density lipoproteins, and poor lymphatic drainage, are believed to enhance this selectivity [12].

The key characteristics of ideal photosensitizers encompass: (1) high quantum yields of singlet oxygen or other reactive oxygen species (ROS); (2) high solubility in water; (3) amphiphilicity, which strikes a balance between water solubility and an affinity for lipophilic cancer cells; (4) enhanced targeting and selectivity facilitated by proper carriers; (5) the prevention of aggregation; (6) minimal photosensitivity; and (7) activation by a proper wavelength of light [13].

The PDT process begins with the administration of photosensitizers, followed by the localized illumination of a specific wavelength of light to activate these agents. This activation leads to the generation of ROS, including singlet oxygen (^1^O_2_), hydrogen peroxide (H_2_O_2_), superoxide ion (O_2_^−•^), and hydroxyl radical (OH^•^), triggering photochemical reactions. These reactions result in direct cancer cell killing through necrosis and apoptosis, as well as indirect vascular damage with hypoxia and starvation, and initiation of an immune response [14]. The extremely short half-lives of ROS in the intracellular environment (<0.1 microsecond) limit their effective range to tumor tissues only [15].

In addition to its cancer selectivity, PDT offers several advantages over conventional cancer treatments. First, it provides a less invasive approach to treatment. Second, PDT can be repeated as needed due to reduced side effects and a lack of resistance mechanisms. Third, PDT easily synergizes with various other cancer treatments, enhancing their effectiveness while preserving other treatment modalities. Fourth, PDT often results in little to no significant scarring or sequelae. Lastly, PDT offers shorter treatment times, increasing patient convenience. As a result, PDT has emerged as an attractive and promising cancer treatment option.

In this study, we review various PDT studies for cancer treatment, categorized by cancer types (lung, head and neck, non-melanoma skin, prostate, and breast cancer). These cancer types have been selected as the ones in which basic research and clinical trials on PDT are most actively underway. The selection of these cancer types is well-justified due to their prevalence, potential responsiveness to PDT, and the availability of recent research and clinical data. We provide an overview of both basic research and clinical trials, summarizing the findings according to the specific photosensitizers utilized. Through this comprehensive review, we aim to highlight the potential and current status of PDT as an innovative and effective therapeutic approach in cancer treatment.

**Figure 1:**
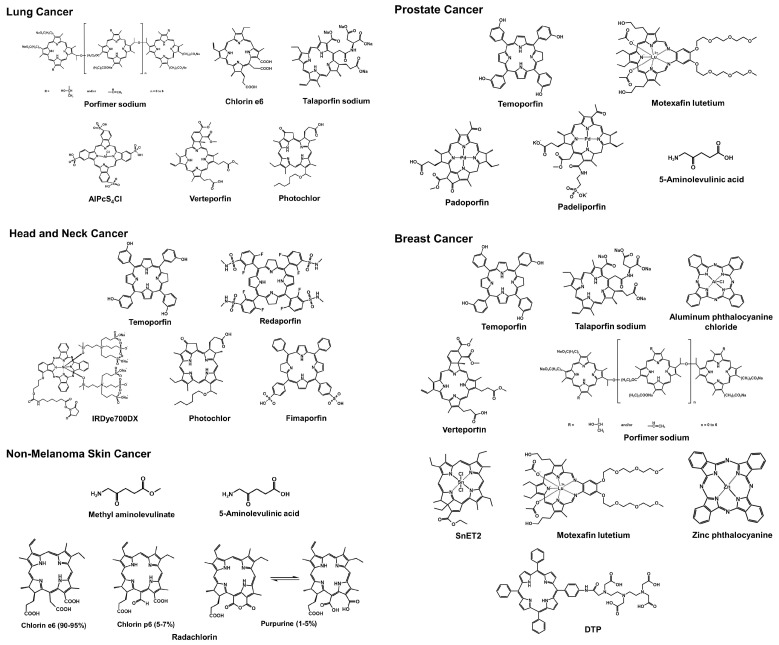
Structural formulas of photosensitizers used in cancer treatment.

Structural formulas of all the photosensitizers introduced in this review are presented in Figure 1. Additionally, photosensitizers utilized in basic research and those currently undergoing clinical trials have been summarized in Table 1 and Table 2, respectively.

## 2. Lung Cancer

Lung cancer is an aggressive disease with a considerable global mortality rate [16]. Each day, approximately 350 individuals succumb to lung cancer, making it nearly 2.5 times deadlier than colorectal cancers, the second most common cause of cancer-related deaths. In the projected 127,070 deaths from lung cancer in 2023, around 103,000 (81%) are expected to be directly attributed to cigarette smoking, while an additional 3560 deaths will be caused by exposure to second-hand smoke [17]. Current treatment methods for lung cancer often exhibit significant systemic toxicity, limited response rates, and poor survival outcomes, emphasizing the urgent need to develop non-invasive and effective therapeutic approaches that cater to the specific needs of lung cancer patients [18].

PDT has emerged as a successful and widely accepted intervention in thoracic oncology for almost three decades. Its simplicity, effective tumor destruction, and compatibility with other cancer treatments have firmly established PDT as an integral part of the modern multidisciplinary approach to thoracic malignancies [19].

### 2.1. Porfimer Sodium

Porfimer sodium, also known as Photofrin^®^, received its initial approval for bladder cancer treatment in Canada in 1993, followed by approval in Japan in 1994 for early stage lung cancer. The U.S. Food and Drug Administration (FDA) granted its first approval in 1995 for advanced obstructive esophageal cancer. Subsequently, in 1998, it was further approved for the treatment of early stage non-small-cell lung cancer [20].

Two studies were conducted to assess the safety and feasibility of PDT for peripheral lung tumors. These studies involved fifteen patients with tumors ranging in size from 1.1 to 3.3 cm. The patients underwent PDT using porfimer sodium with laser light (630 nm) applied at 200 J/cm^2^. The results showed that there were no deaths or discontinuations due to adverse events. Eight patients experienced photosensitivity reactions, most of which were mild to moderate. In the non-resection study, one patient achieved a complete response at 6 months. In the resection study, one patient had no evidence of tumor at resection, three had 40–50% tumor cell necrosis, two had 20–35%, and four had 5–10%. Based on these results, the authors concluded that PDT is a feasible and relatively safe treatment for peripheral lung tumors [21].

In a clinical trial (NCT03344861), the safety and feasibility of PDT for peripheral lung tumors were further assessed. The trial consisted of two studies: a non-resection study and a resection study. In the non-resection study, five patients with peripheral lung tumors underwent PDT using porfimer sodium and navigational bronchoscopy. After six months of clinical follow-up, one patient achieved a complete response, indicating the absence of any detectable tumors. In the resection study, ten patients underwent PDT, followed by a surgical removal of the treated tumors. One patient had no evidence of tumor at resection, representing a complete response. Three patients showed 40–50% tumor cell necrosis, two had 20–35% necrosis, and four had 5–10% necrosis, indicating varying degrees of tumor response to PDT. Eight out of fifteen patients experienced photosensitivity reactions, which were mostly mild to moderate in severity. Overall, this trial demonstrated that PDT for peripheral lung tumors is feasible and safe, with no deaths or treatment discontinuations.

Additionally, a case study was conducted for the treatment of primary non-small-cell lung cancer using Photofrin^®^-PDT. Within 48 h of receiving an intravenous injection of Photofrin^®^ at a dosage of 2 mg/kg, a single illumination session lasting 500 s and delivering 200 J/cm^2^ at a wavelength of 630 nm was targeted toward the solitary peripheral lesion. The procedure was carried out without any complications. After 30 days, a lobectomy was conducted as planned, and the pathology results showed necrosis and no viable tumor remaining. At a 90-day follow-up, the patient was in good health with no signs of disease [22].

Several ongoing clinical trials are investigating the use of Photofrin^®^-PDT for the treatment of lung cancer (NCT03678350, NCT03735095, and NCT04836429). These trials aim to further evaluate the safety and efficacy of PDT with Photofrin^®^ in different settings and patient populations.

In summary, porfimer sodium-PDT shows promising potential as a treatment option for lung cancer, particularly for peripheral lung tumors. The available research and ongoing clinical trials continue to contribute valuable insights into its effectiveness and safety, paving the way for potential advancements in PDT for lung cancer patients.

### 2.2. Chlorin e6

Chlorin e6 (Ce6), a second-generation photosensitizer approved by the FDA [23], is widely utilized in PDT due to its efficient production of singlet oxygen, ability to be excited using near-infrared light for deep tissue penetration, and capability for fluorescence imaging. However, Ce6 faces challenges such as limited water solubility and nonspecific accumulation at tumor sites, which significantly hinder its therapeutic effectiveness [24,25].

In a notable in vivo study, Gd^3+^- and Ce6-loaded single-walled carbon nanohorns (Gd-Ce6@SWNHs) demonstrated strong immune adjuvant properties and high tumor targeting efficiency. The researchers employed a sequential approach, first using PDT with a 650 nm wavelength and low laser power, followed by photothermal therapy (PTT) with an 808 nm wavelength and higher power intensity in a spontaneous lung metastasis model using an orthotopic breast cancer cell line (4T1 cell). The combination therapy resulted in the complete elimination of spontaneous pulmonary metastases in 80% of the mice, and no recurrence was observed for 18 months. The sequential PDT + PTT treatment eradicated primary tumors, induced diverse damage-associated molecular patterns (DAMPs), and elicited a complementary and synergistic immune response [26].

Talaporfin sodium is a Ce6 derivative, and its chemical structure is the tetra-sodium salt of mono-L-aspartyl Ce6 [20]. As a second-generation hydrophilic photosensitizer, talaporfin sodium (Talaporfin^®^) is associated with less skin photosensitivity compared to Photofrin^®^ [27].

A clinical trial of Talaporfin^®^-PDT for c-stage IA peripheral lung cancer demonstrated promising results. The trial revealed that PDT is a viable and non-invasive treatment option for early stage peripheral lung cancers. In this trial, a cutting-edge laser probe called the composite-type optical fiberscope (COF) was introduced, showcasing its ability to enable precise laser irradiation and the simultaneous visualization of cancerous lesions. Talaporfin sodium was administered intravenously at a dose of 40 mg/m^2^, four hours prior to the PDT procedure. The COF was carefully inserted into the cancer lesion, allowing for the real-time visualization of the tumor, which was then targeted with a red laser (664 nm) for irradiation. Encouragingly, at the three-month follow-up after PDT, four patients exhibited a complete response, and remarkably, this response persisted in those same four patients even at the one-year mark following PDT [28].

A retrospective study was conducted to evaluate the safety of Talaporfin^®^-PDT as a day treatment for central early stage lung cancer (CELC). The optimal conditions for high safety and efficacy were determined as the intravenous administration of Talaporfin^®^ (40 mg/m^2^) followed by laser irradiation (100 J/cm^2^) after 4 h, using a diode laser apparatus. Talaporfin^®^ was administered between 10 am and 12 pm on the day of treatment, with a dose of 40 mg/m^2^ for all patients. No severe adverse events were observed among the remaining 11 patients (15 treatments), although mild and temporary photosensitivity reactions were reported in a small number of cases. The study concluded that Talaporfin^®^-PDT can be safely performed as a day treatment for CELC patients [29].

An approval was granted in Japan in 2004 for the use of Laserphyrin^®^, another derivative of Ce6, in PDT for early stage lung cancer [30]. A study was conducted to evaluate the effectiveness of Laserphyrin^®^-PDT in treating advanced lung cancer patients with airway stenosis. Since surgical options are often limited for patients with central airway stenosis due to lung cancer, palliative interventions are frequently required. Twelve patients were enrolled in the study, receiving intravenous administration of Laserphyrin^®^ (40 mg/m^2^) followed by PDT using a 664 nm wavelength laser beam. Subsequently, chemotherapy was administered on the same day or the day after PDT. The results showed that the median stenosis rates before treatment, one-week post-treatment, and one-month post-treatment were 60%, 15%, and 15%, respectively. The mean survival time was 9.3 months, with a median survival time of 5.9 months, and the overall 1-year survival rate was 30.0%. PDT demonstrated no associated morbidity or mortality, and patients experienced improved symptoms and quality of life as early as one week after treatment. The study concluded that PDT effectively reduced airway stenosis in patients with advanced lung cancer [31].

### 2.3. AlPcS_4_Cl

AlPcS_4_Cl, also known as aluminum (III) phthalocyanine chloride tetra-sulfonate, is a compound with desirable properties as an efficient photosensitizer. Extensive studies have been conducted, revealing its excellent photo-stability and amphipathic nature [32]. Despite its potential, AlPcS_4_Cl has not yet been approved for cancer treatment.

An in vitro study was conducted to evaluate the effective dose responses of AlPcS_4_Cl on A549 cells (human lung adenocarcinoma cells). This study investigated the photochemical characteristics of AlPcS_4_Cl, its internalization into lung cancer cells, intracellular distribution, and photodynamic effects on lung cancer. The results demonstrated that AlPcS_4_Cl acts as a stable photosensitizer, localizing in intracellular organelles, including the mitochondrion and lysosomes. Consequently, AlPcS_4_Cl showed promising effectiveness in treating lung cancer [33].

Furthermore, researchers conducted another in vitro study to assess the efficacy of AlPcS_4_Cl as a photosensitizer for eradicating lung cancer stem cells (CSCs) through PDT. A549 cells were irradiated with a 673.2 nm diode laser (10 J/cm^2^) after the addition of AlPcS_4_Cl. The results indicated that the intracellular localization of AlPcS_4_Cl induced cell death, with the photosensitizer accumulating in the cytosol and vital organelles. This led to the generation of free radicals upon photoactivation, resulting in cellular destruction. The study observed the morphological features of apoptosis and necrosis, reduced cell proliferation and viability, and significant mitochondrial damage, ultimately leading to apoptotic cell death [34].

Moreover, investigations were carried out to examine the effects of AlPcS_4_Cl-gold nanoparticle (AuNP) and AlPcS_4_Cl-AuNP-antibody (Ab) on lung CSCs. These cells were obtained from a side population of A549 cells and were cultured for attachment for 4 h. Subsequently, the cells were irradiated with 673.2 nm light at a fluence of 10 J/cm^2^, followed by the addition of the photosensitizer. The results demonstrated that these treatments resulted in significant cell toxicity and death in lung CSCs, surpassing the effects of free AlPcS_4_Cl alone. Furthermore, the use of the nano-bioconjugate further enhanced the PDT effects, leading to the destruction and eradication of lung CSCs. Conjugating AlPcS_4_Cl to AuNPs and anti-CD133 improved the uptake of AlPcS_4_Cl in lung CSCs, effectively delivering the drug compared to AlPcS_4_Cl alone. This enhanced delivery led to increased cytotoxicity, apoptosis, and reduced proliferation and viability of lung CSCs, indicating the potential of active AuNP-Ab targeting to improve the outcomes of PDT treatment for lung cancer [32].

These findings underscore the promise of AlPcS_4_Cl as a photosensitizer for lung cancer treatment and suggest its potential application in targeted and effective PDT strategies for this devastating disease.

### 2.4. Verteporfin

Verteporfin, known by the trade name Visudyne^®^, is an FDA-approved treatment for choroidal neovascularization but has not yet been approved for lung cancer. Visudyne^®^ is a second-generation photosensitizer that incorporates verteporfin into liposomes using a repeated freeze–thaw method. This liposomal formulation enhances specific accumulation in neovascularization and binding with apolipoprotein [35]. Compared to verteporfin alone, Visudyne^®^ exhibits increased photosensitizer activity and stronger anti-angiogenic properties, resulting in an enhanced inhibition of tumor growth.

However, it is worth noting that PDT efficacy may be influenced by hypoxia [18,36]. In an in vivo study, the impact of Perftoran^®^ administration, an oxygen carrier, on the efficiency of Visudyne^®^/PDT and its effect on the hypoxia pathway in a murine lung cancer model were investigated. When the oxygen levels in the tumor microenvironment are insufficient, the efficacy of PDT can be diminished or blocked. To address this issue, one group of lung cancer-bearing mice received an intraperitoneal injection of Visudyne^®^ at a dose of 4 mg/kg body weight for PDT, while another group received an intravenous injection of Perftoran^®^ (5% emulsion per kg body weight) before Visudyne^®^/PDT. The results demonstrated that when Perftoran^®^ was administered before Visudyne^®^/PDT, it acted as an oxygen carrier and significantly improved the treatment outcome. This combination treatment led to necrosis in the bronchiolar epithelium, with the accumulation of necrotic debris and late apoptosis. Additionally, this combination effectively suppressed hypoxia levels, inhibited HIF-1α concentration, and reduced vascular endothelial growth factor (VEGF) expression [18].

These findings indicate that combining Perftoran^®^ with Visudyne^®^/PDT holds promise as a potential therapeutic strategy for lung cancer. By addressing the issue of hypoxia in the tumor microenvironment, this combination approach may enhance the efficacy of PDT and improve treatment outcomes for lung cancer patients. Further research and clinical trials are warranted to validate and refine this treatment approach for potential future clinical applications.

### 2.5. 2-[1-Hexyloxyethyl]-2-Devinyl Pyropheophorbide-a (HPPH)

2-[1-Hexyloxyethyl]-2-devinyl pyropheophorbide-a (HPPH), also known as photochlor [20] is an investigational compound that has not yet been approved for cancer treatment [37]. In a phase I study, researchers aimed to determine the maximum tolerated light dose at a fixed photosensitizer dose and assess the effectiveness of this treatment approach. Seventeen patients with 21 carcinomas in situ (CIS) and microinvasive cancer (MIC) lesions were enrolled in the study. These patients received HPPH with escalating light doses ranging from 75 to 150 J/cm^2^. Among the participants, thirteen had one lesion treated, while four had two lesions treated with the same light dose. HPPH was administered through intravenous infusion, and vital signs were closely monitored post-infusion.

Based on the study findings, an HPPH dose of 4 mg/m^2^ was found to be effective, and the light dose should not exceed 125 J/cm^2^ for optimal outcomes. The results indicated that PDT with HPPH can be safely used for the treatment of CIS/MIC, offering potential effectiveness comparable to porfimer sodium but with a shorter light precaution duration [38].

These findings suggest that HPPH shows promise as a potential photosensitizer for PDT in the treatment of early stage cancers such as CIS/MIC. However, further research and clinical trials are necessary to validate its safety and efficacy in a larger patient population and to assess its potential for use in other cancer types and stages. With continued investigation, HPPH may become a valuable addition to the arsenal of PDT options for cancer treatment.

## 3. Head and Neck Cancer

Head and neck cancer constitutes approximately 4% of all cancers in the US and had around 562,328 global diagnoses in 2020, with an estimated 15,400 deaths predicted for 2023 in the US alone (11,210 men and 4190 women) [17].

Early stage head and neck cancer is generally managed with radiotherapy and/or surgery, but advanced carcinoma is treated with a combination of chemotherapy and radiation. Surgical excision requires a wide area, which may cause functional disorders and speech and swallowing problems. Additionally, radiotherapy can lead to adverse effects such as xerostomia and trismus. Due to the complex features of the head and neck region, the treatment of head and neck cancer can significantly impact the quality of life (QOL) [39].

PDT is locoregionally activated, and tissue perforation is limited, making it appropriate for the treatment of extensive skin lesions or sensitive lesions, such as head and neck cancer [40]. PDT has shown effectiveness that is comparable to primary surgical excision for early stage squamous cell carcinoma (SCC) of the oral cavity [41].

### 3.1. Temoporfin

Temoporfin, also known as m-tetrahydroxyphenylchlorin (mTHPC) or Foscan^®^, was approved by the European Medicines Agency (EMA) for the palliative treatment of advanced head and neck squamous cell carcinoma in 2001. An in vitro study was conducted to test the hypothesis that combining Foscan^®^-mediated PDT with fenretinide improves carcinoma cell killing. SCC19 cells (human head and neck squamous cell carcinoma cells) were treated with Foscan^®^ and N-(4-hydroxyphenyl) retinamide (HPR), a desaturase inhibitor fenretinide, in the growth medium. The cells were cultured with 0.06 μM Foscan^®^ overnight, followed by the addition of 2.5 μM HPR. Immediately after HPR addition, the cells were irradiated with 400 mJ/cm^2^ of red light (μmax ~ 670 nm) and cultured for the appropriate time. The results showed that PDT with HPR treatment increased the accumulation of non-ceramide C-16-dihydroceramide and enhanced mitochondrial depolarization in SCC19 cells, indicating that combining Foscan^®^-mediated PDT with fenretinide (HPR) improves the apoptosis and anti-tumor efficacy of PDT [42].

A clinical trial was performed to demonstrate the clinical benefit of PDT after surgery with close or positive resection margins in head and neck cancer. After 54 patients underwent surgery, mTHPC-mediated PDT was conducted. mTHPC (0.15 mg/kg) was administered, followed by an illumination of the target area 96 h later using a 652 nm red laser light with a dose of 20 J/cm^2^. The progression-free survival rate was 30%, the disease-free survival rate was 28%, and the overall survival rate was 51% at 2 years. Notably, a significantly better disease-free survival was observed with a time interval between surgery and PDT of at least 6 weeks. Overall, PDT can be applied as adjuvant therapy in head and neck cancer after surgery with tumor-positive resection margins, but the clinical benefits are not yet fully established [43].

Recently, an exploratory study was published, analyzing the effect of PDT with temoporfin in the head and neck area on the QOL of patients. The study reported an improved QOL in all 38 patients who had head and neck cancer after mTHPC-PDT. All patients treated for various head and neck pathologies using mTHPC-PDT completed the questionnaire during an average 56-day follow-up. Pain after PDT was reported as the main trouble, which is a common adverse effect. In the 4 weeks after PDT, improvements were observed in swallowing, speaking, visual symptoms, and breathing, and in the following weeks, enhancements in daily life activities and social life were reported. In some head and neck cancer patients, mTHPC-PDT offered improvements in QOL scores comparable to other treatments [39].

### 3.2. Redaporfin

Redaporfin, a synthetic bacteriochlorin, is an anticancer agent also known as LUZ11 or F-2BMet [20,44]. While redaporfin is approved as an orphan drug by the EMA for the treatment of cholangiocarcinoma, it is not yet approved for the treatment of head and neck cancer [45]. In a preclinical trial, immune responses after vascular redaporfin-PDT were investigated. Mice were injected with 350,000 CT-26 cells subcutaneously and treated with redaporfin-vascular-PDT. They received redaporfin at a dose of 0.75 mg/kg, followed by irradiation with 748 nm light at a fluence of 50 J/cm^2^. The results showed that extensive tissue damage was induced by redaporfin-vascular-PDT at the irradiated tumor site, and the treatment caused acute local inflammation marked by neutrophilia and IL-6 expression, observed within 1 day after PDT [46].

Phase I/IIa clinical trials were conducted to determine the anti-tumor effect, tolerability, and pharmacokinetics of redaporfin in patients with advanced head and neck cancers. The patients received increasing single doses of redaporfin intravenously at intervals of about 21 days, with the following doses administered: 0.05 mg/kg (*n* = 6), 0.1 mg/kg (*n* = 4), 0.25 mg/kg (*n* = 2), 0.5 mg/kg (*n* = 4), 0.75 mg/kg (*n* = 6), and 1.0 mg/kg (*n* = 1). After redaporfin administration, a laser light at 749 ± 3 nm with a fluence of 50 J/cm^2^ was irradiated to the lesion area, and the light dose that was safe and caused a necrosis of the tumor surface was also evaluated. Photodynamic effects were observed in all patients who received more than 0.5 mg/kg of redaporfin. Patients who completed the final PDT session showed a complete tumor necrosis reaction at the lesion area. The half-life of redaporfin was measured to be 19 h, and it was relatively quickly eliminated from the body. The effective dose of redaporfin was determined to be 0.75 mg/kg, and redaporfin-PDT demonstrated anti-tumor effects and safety in patients with advanced head and neck cancer.

A case report demonstrated the successful treatment of resistant head and neck squamous cell carcinoma using redaporfin-PDT in combination with nivolumab, an immune checkpoint inhibitor. The patient had a large tumor in the mouth despite undergoing radiotherapy and surgery. After receiving an injection of redaporfin at a dose of 0.75 mg/kg, the tumor area was illuminated with 749 nm laser light at a fluence of 50 J/cm^2^. Redaporfin-PDT led to the elimination of all visible tumor, and the sequential use of nivolumab, the immune checkpoint inhibitor, resulted in a continuous complete response [45].

### 3.3. IRDye700DX

The IRDye700DX, also known as IR700, is a water-soluble photosensitizer and is currently approved in a formulation conjugated with cetuximab. Akalux^®^ (ASP-1929, RM-1929, cetuximab saratolacan) was developed by Rakuten Medical Japan and was approved by Japan in September 2020 for the treatment of unresectable locally advanced or recurrent head and neck carcinoma. It is the first antibody–photosensitizer drug that conjugates IR700, a water-soluble photosensitizer, with cetuximab, an epidermal growth factor receptor (EGFR) receptor-targeting antibody [47].

In an in vitro study, the format of targeted PDT using nanobodies (7D12, R2, 7D12-9GB) as targeting agents and a water-soluble IRDye700DX was found to be selective in EGFR overexpression cells. Three human cell lines were used: squamous cell carcinoma of the tongue OSC-19-luc2-cGFP, cervical cancer cell line HeLa, and colorectal cancer cell line SW620. These cell lines exhibited different levels of EGFR expression (EGFR expression rate: OSC > HeLa > SW620). The nanobody–IR700 conjugates were applied to each cell line and exposed to 690 nm light (10 J/cm^2^) after 30 min. The study showed that the higher the expression level of EGFR, the higher the fluorescence intensity of 7D12-IR700 and 7D12-9G8-IR700, indicating the specificity of 7D12-IR700 and 7D12-9G8-IR700 for binding to EGFR (OSC > HeLa > SW620). Additionally, the water-soluble IR700 had no cell binding ability unless it was conjugated with the EGFR-targeting nanobody [48].

A phase I/IIa open-label, multicenter study was conducted to confirm the safety and effectiveness of photoimmunotherapy with RM-1929 in heavily pretreated recurrent head and neck squamous cell carcinoma patients. Nine patients participated in the dose-finding part, and thirty patients participated in the safety and effectiveness part. No dose-limiting toxicities were observed in the dose-finding group. In the other group, patients were administered 640 mg/m^2^ of RM-1929 with 50 J/cm^2^ or 100 J/cm^2^ light dose (690 nm). Adverse effects were mostly mild to moderate, but 19 patients experienced grade 3 adverse effects. Overall, photoimmunotherapy with RM-1929 was well tolerated, and responses and survival were clinically meaningful in recurrent head and neck cancer patients [49].

Currently, a global phase III clinical trial is underway using an antibody against EGFR conjugated to IR700 molecule (ASP-1929) in patients with recurrent head and neck cancers who have failed at least two lines of therapy (NCT03769506).

### 3.4. Photochlor

This compound is also known as HPPH [20]. As of now, it is not approved for the treatment of cancer [37]. Phase Ib clinical trials using HPPH-PDT have been studied for the treatment of early stage larynx cancer. This study aimed to assess the safety and preliminary treatment responses of HPPH-PDT in early stage larynx cancer. A total of 29 patients with high-risk dysplasia and SCC of the larynx were enrolled. All patients received an intravenous injection of HPPH at a dose of 4 mg/m^2^, 24 to 26 h before light illumination. Initially, light doses of 50, 75, and 100 J/cm^2^ were delivered to three patients each. However, in two other patients, illumination at 125 J/cm^2^ resulted in dose-limiting toxicities, leading to a reduction in light intensity to 100 J/cm^2^. Ultimately, the maximum tolerated dose was determined to be 100 J/cm^2^, and a total of 30 lesions in 29 patients were treated. The most common adverse reaction was temporary hoarseness. Among patients with T1 SCC, a complete response rate of 82% was observed using HPPH-PDT at the maximum tolerated dose [50].

Another clinical trial was performed to assess the safety and treatment response of HPPH-PDT for early squamous cell carcinoma of the head and neck. Forty patients were administered 4 mg/mm^2^ of HPPH, and light at doses ranging from 50 to 140 J/cm^2^ were illuminated (140 J/cm^2^). After 12 weeks, pathologic tumor responses were evaluated. Common side effects included edema and pain at the treatment site. The complete response rates were 82% for SCC lesions [51].

### 3.5. Fimaporfin

Fimaporfin, also known as disulfonated tetraphenyl chlorine (TPCS2a), is currently not approved for the treatment of cancer. A phase I clinical trial was conducted to evaluate the safety and tolerability of dose-escalating TPCS2a for mediating the photochemical internalization of bleomycin in patients with advanced and recurrent solid tumors, including head and neck cancer. A total of 22 patients were enrolled in this trial, most of whom had squamous cell carcinoma of the head and neck, and all had previously undergone surgery and chemotherapy for the lesion.

In the trial, four days after an intravenous administration of TPCS2a, 15,000 IU/m^2^ of bleomycin was also administered intravenously. After 3 h, the surface of the target tumor was exposed to 652 nm light at a dose of 60 J/cm^2^. The initial dose of TPCS2a was 0.25 mg/kg, and subsequent doses were escalated in three patients to 0.5, 1.0, and 1.5 mg/kg.

During the 3-month follow-up period, twelve patients completed the study. The maximum tolerated dose of TPCS2a was determined to be 1.0 mg/kg. However, strong anti-tumor effects were observed at all doses, including the starting dose of 0.25 mg/kg. Side effects related to photochemical internalization included local inflammation and systemic skin photosensitization caused by TPCS2 [52].

## 4. Non-Melanoma Skin Cancer

Skin cancers can be categorized into two main groups: melanoma and non-melanoma [40]. Among non-melanoma tumors, the most common types are basal cell carcinoma (BCC) and SCC. Among non-melanoma skin cancers, BCC is the most prevalent, with approximately 85% of all BCCs occurring in the head and neck region. Although BCC rarely metastasizes, it should be treated promptly to prevent local tissue destruction [53].

For specific non-melanoma skin cancers, topical PDT has emerged as an outstanding non-invasive treatment option for diverse and large lesions. PDT, when administered with standardized protocols, yields better cosmetic results and improved therapeutic effects compared to conventional treatments, particularly in cases of superficial BCC. The treatment is generally well-tolerated, although patients may experience tingling pain during the procedure [54].

### 4.1. Methyl Aminolevulinate

Metvix^®^ (methyl aminolevulinate, MAL) has been FDA-approved for the treatment of actinic keratosis (AK) and BCC worldwide. Several ongoing clinical trials are investigating the use of MAL-based PDT (MAL-PDT) for the treatment of AK, SCC, and BCC.

In an in vitro study, the efficacy of MAL-PDT in resistant cells (HSC-1 cells) was enhanced by using epigallocatechin gallate (EGCG), which showed promising results for the treatment of difficult-to-treat skin cancer. The study involved culturing human skin cell line HSC-1, a derivative of squamous cell carcinomas, and incubating it with 2 mM MAL. The cells were then irradiated with 630 nm red light (4 J/cm^2^). Another group of HSC-1 cells was incubated with 2 mM MAL and EGCG (10–80 μM) to assess the effectiveness of EGCG in PDT-resistant cells. These cells were also irradiated with 630 nm red light (4 J/cm^2^). The results indicated that PDT-resistant HSC-1 cells exhibited low levels of protoporphyrin IX and ROS, resulting in a low rate of cell death. However, the addition of EGCG improved the effectiveness of MAL-PDT, leading to increased levels of protoporphyrin IX and ROS, and achieving 100% cell death [55].

In a clinical trial, the efficacy of two different PDT-based therapies was evaluated for high-risk facial nodular BCC. A total of 32 patients with Fitzpatrick’s skin types II (*n* = 20) and III (*n* = 12) were enrolled. The patients were randomly assigned to receive either conventional PDT or ablative fractional laser-primed PDT (AFXL-PDT). In the AFXL-PDT group, 16% Metvix^®^ cream was applied after AFXL treatment, followed by irradiation at 37 J/cm^2^ light dose for 8 min. After three months, all 16 patients treated with AFXL-PDT showed complete responses, while the complete response rate was slightly lower (88%) in the conventional PDT group. However, at the 12-month follow-up, the tumor clearance results were similar in the AFXL-PDT (63%) and PDT (56%) groups [56].

Another phase III trial was conducted to evaluate the safety and efficacy of BF-200 aminolaevulinic acid (Ameluz^®^) gel compared to Metvix^®^ cream for the treatment of nonaggressive BCC with PDT. A total of 281 patients were enrolled in the trial and randomly applied either Ameluz^®^-PDT or Metvix^®^-PDT. Of the Ameluz^®^-PDT patients, 93.4% showed complete response, while the Metvix^®^-PDT group had 91.8% complete response. The treatment of nonaggressive BCC with Ameluz^®^-PDT was found to be effective and better tolerated than Metvix^®^-PDT, indicating lower recurrence rates after the 12-month follow-up [57].

In another phase I trial, the efficacy of one session of ablative fractional laser-primed PDT (AFL-PDT) was compared to two sessions of MAL-PDT for the treatment of microinvasive SCC. A total of 45 patients participated in the trial, with 21 patients receiving one session of AFL-PDT and 24 patients receiving two sessions of MAL-PDT with a 7-day interval between sessions. The results after three months showed a complete response rate of 84.2% with AFL-PDT and 52.4% with MAL-PDT. Moreover, the results were maintained significantly for the 24-month follow-up, with a higher recurrence rate observed with MAL-PDT (63.6%) compared to AFL-PDT (12.5%) over the two-year period. Cosmetic outcomes, adverse events, and pain intensity were not significantly different between AFL-PDT and MAL-PDT [58].

Recently, a phase III clinical trial was conducted to observe the complete response and cosmetic results of PDT with Metvix^®^ cream in patients with high-risk BCCs. Patients were applied Metvix^®^ cream 160 mg/g, followed by illumination using non-coherent light with a fluency of 75 J/cm^2^ for up to 13 weeks.

### 4.2. 5-Aminolevulinic Acid

5-Aminolevulinic acid (5-ALA, Levulan^®^) has received FDA approval for the treatment of various diseases, including cutaneous superficial and nodular BCC, SCC, and AK [59].

An in vitro study investigated the anti-tumor efficacy of PDT using 5-ALA-gold nanoparticles (5-ALA-GNPs) in cutaneous squamous cancer cells (A431 cells). The cells were incubated with 5-ALA (2, 4, and 8 mM) or 5-ALA-GNPs (2, 4, and 8 mM), and then exposed to light at 621 nm. Compared to PDT with 5-ALA alone, the use of GNPs conjugated to 5-ALA improved cell apoptosis and singlet oxygen generation in A431 cells. It also enhanced the inhibition of cell invasion, cell migration, and Wnt/β–catenin signaling activities. The results indicate that 5-ALA-GNP improves the anti-tumor effectiveness of PDT in A431 cells, suggesting its potential to enhance treatment outcomes for patients with cutaneous squamous cell carcinoma [60].

A phase III clinical trial was conducted to evaluate whether twofold 5-ALA-PDT is superior to the established MAL-PDT. A total of 162 patients participated in this trial, with 82 patients receiving 5-ALA 20% cream at the BCC area and another group of 80 patients receiving Metvix^®^ cream at the BCC area. The patients were randomly assigned to either twofold ALA-PDT or established MAL-PDT. The treatment involved light delivery with specific fluence to the lesions at designated time intervals. After one year, six treatment failures occurred using ALA-PDT, while 13 failures took place using MAL-PDT. Patients treated with ALA-PDT also experienced side effects more frequently. The probability of treatment success after one year was 92.3% for ALA-PDT and 83.4% for MAL-PDT. Although the difference between the two treatment groups was not statistically significant, the twofold ALA-PDT method showed a lower recurrence rate [61].

Currently, a phase II trial is underway to determine the time to maximum expression of immune checkpoint molecules and the ratio of cytotoxic T cells to regulatory T cells in BCC and peri-tumoral stroma after ALA-PDT, as compared to untreated tumors (NCT05020912). Another phase III trial is also in progress to test the safety and efficacy of PDT with Ameluz^®^, a nanoscale lipid vesicle formulation, performed with PDT-lamp BF-RhodoLED^®^, compared to placebo treatment for superficial BCC (NCT03573401).

### 4.3. Radachlorin

Radachlorin^®^ has been approved by the Ministry of Health of the Russian Federation (MHRF) for the treatment of skin cancer [62]. A study involving 30 patients with BCC with various histological types and clinical forms was conducted to evaluate the efficacy of PDT with Radachlorin^®^.

For the treatment, a dose of 1.75–3.50 mg/cm^2^ of Radachlorin^®^ was injected into the tumor (0.5–1.0 mL of Radachlorin^®^ solution per 1 cm^2^ of the lesion area). The time interval between Radachlorin^®^ injection and irradiation was set to 10–15 min. On the second to seventh day after PDT, hemorrhagic necrosis of the tumor was observed with a distinctly outlined border, corresponding to the irradiated area. After 7 days, a scab formed over the tumor area, which eventually peeled away after 1–2 months. Subsequently, a whitish scar formed on the treated area [63].

## 5. Prostate Cancer

Prostate cancer is the second most commonly occurring cancer in men and the fourth most common cancer overall. In 2020, more than 1.4 million new cases of prostate cancer were reported worldwide, and an estimated 268,490 new cases are expected to be reported in the US alone in 2022 [64]. Treatment options for prostate cancer vary based on the stage and grade of the cancer and patient-specific factors, including age and comorbidities. For patients with low-risk, localized cancers or those who are elderly and/or have other serious health conditions, active monitoring is generally recommended instead of immediate treatment. In cases of advanced prostate cancer, treatment options may include radiotherapy, chemotherapy, androgen deprivation therapy, bone-directed therapy, or a combination of these modalities. However, surgery and radiotherapy are associated with significant risks of physical disabilities, such as erectile dysfunction, urinary incontinence, and intestinal complications [64].

PDT holds promise as a potential major treatment for prostate cancer. However, the current PDT methods have limitations and unpredictability. These weaknesses can be overcome by optimizing parameters, including photosensitizer dose and light dose [65].

### 5.1. Temoporfin

Foscan^®^ is not approved for the treatment of prostate cancer; however, it is approved for head and neck cancer. Several preclinical and clinical trials using Foscan^®^ are ongoing for the treatment of prostate cancer. In vitro studies assessed the photodynamic characteristics of FosPeg^®^, a PEGylated liposomal formulation of mTHPC, using LNCaP human prostate cancer cells. FosPeg^®^ demonstrated higher intracellular absorption at any concentration compared to Foscan^®^. Regarding PDT effectiveness, FosPeg^®^ exhibited a more severe cytotoxicity at any light dose and concentration than Foscan^®^. A FosPeg^®^ concentration of 0.22 μM and a light dose of 180 mJ/cm^2^ were sufficient to achieve half of the tumor cells’ death 24 h after PDT, whereas Foscan^®^ required a concentration of 1.8 μM to achieve the same result. The PEGylated liposomal formulation of mTHPC showed greater advantages as a photosensitizer compared to Foscan^®^ due to enhanced intracellular absorption and photodynamic activity [66].

In a phase I clinical trial, PDT with mTHPC was utilized to treat locally recurrent prostate carcinoma after radiotherapy. Fourteen men with recurrent prostate carcinoma after radiotherapy participated in the trial. Three days after intravenous injection of 0.15 mg/kg mTHPC, treatment with light at 652 nm wavelength was delivered via optical fibers inserted percutaneously. All patients tolerated PDT well, with up to 91% of the prostate cross-section showing necrosis, and five patients had no viable cancer cells on post-treatment biopsies [67].

Another phase I clinical trial was conducted to treat early stage prostate cancer. mTHPC (0.15 mg/kg) was intravenously injected into six patients with early stage prostate cancer, and 2–5 days later, light was irradiated to the site of the tumor lesion using optical fibers inserted through transperineal needles. As a result, the prostate-specific antigen (PSA) decreased down to 67%. Initially, uneven necrosis and edema were observed, but these issues resolved over a period of 2 months [68].

### 5.2. 5-Aminolevulinic Acid

5-ALA is not approved for the treatment of prostate cancer; however, it is approved for BCC. Several preclinical and clinical trials using ALA and its derivatives are ongoing for the treatment of prostate cancer. In human prostate cancer cell lines (PC-3, 22Rv1, DU145, and LNCaP), predictors of the effectiveness of ALA-PDT were studied. Each prostate cancer cell line exhibited different results for intracellular accumulation of protoporphyrin IX (PpIX) and cytotoxicity via ALA-PDT. PC-3 and LNCaP cells showed a high accumulation of PpIX and high sensitivity to ALA-PDT, while 22Rv1 and DU145 cells showed the opposite results. There was a negative correlation between the cytotoxicity of ALA-PDT and the accumulation of PpIX. In vitro studies indicated that the sensitivity of ALA-PDT depends on the expression level of the ATP-binding cassette transporter subfamily G2 (ABCG2) transporter dimer, which can be used as a predictor of treatment response. 22Rv1 cells showed a high expression of the ABCG2 transporter, while PC-3 cells showed low expression. Consequently, tumor atrophy via ALA-PDT was greater in PC-3 cells than in 22Rv1 cells. The study demonstrated that the ABCG2 transporter is a useful predictor of PDT with ALA in human prostate tumor cells [69].

A phase I trial was conducted to demonstrate that PDT with 5-ALA-induced PpIX of prostate carcinoma is a simple and safe procedure. In 14 patients who underwent radical retropubic prostatectomy after oral use of 5-ALA (20 mg/kg), a localization of 5-ALA-induced PpIX was observed in the prostate glands. Fluorescence of PpIX at 635 nm was detected in tumor cells and not in stromal or epithelial tissue. As a result, five patients were treated with PDT using 5-ALA. Among these five patients, three were treated using interstitial transurethral PDT, and two underwent PDT through the perineum. In both treatments, a laser light of wavelength 633 nm was used. On average, a 55% decrease in PSA was observed after 6 weeks in patients using interstitial transurethral PDT, and patients treated with PDT through the perineal approach had a 30% decrease in PSA. No complications were reported [70].

A phase II trial related to the diagnosis of prostate carcinoma was also conducted. The results of this clinical trial indicated that photodynamic diagnosis with 5-ALA-induced PpIX during radical prostatectomy might be an effective and feasible method for reducing the proportion of positive surgical margins [71].

### 5.3. Padoporfin

Padoporfin (WST9, Tookad^®^) is approved in the EMA for the treatment of prostate cancer [72]. It is a bacteriochlorophyll-derived photosensitizer that exhibits powerful light absorbance in the near-infrared part at 763 nm, allowing for deep tissue penetration [20].

In preclinical studies, Tookad^®^-PDT demonstrated efficacy and good tolerability as a minimally invasive therapy for disseminated and locally advanced small-cell carcinoma of the prostate. Human small-cell carcinoma of the prostate was grafted into male CD1-nude mice in three relevant anatomical regions (subcutaneous, intraosseous, and orthotopic within the mice prostate microenvironment). After intravenous injection of 4 mg/kg Tookad^®^, light therapy with a wavelength of 650–800 nm was immediately applied. The control groups included untreated animals, animals treated with light alone, and animals injected with Tookad^®^ alone. Subcutaneous tumors showed a complete response within 28–40 days, resulting in an overall long-term cure rate of 69%. Intratibial WISH-PC2 lesions showed tumor elimination in 50% of the cases within 70–90 days. Similar results were observed in the orthotopic model [73].

In a phase I clinical trial to evaluate the safety and treatment response for recurrent prostate cancer following definitive radiation treatment, Tookad^®^-PDT was used. This trial demonstrated the application of vascular-targeted PDT (VTP) with Tookad^®^, where tumors are destroyed by injuring their blood supply when activated with 763 nm light. Six patients received increasing drug doses of 0.1 to 2 mg/kg at a light dose of 100 J/cm^2^. Three patients responded at a dose of 1 mg/kg, and two patients responded at a dose of 2 mg/kg. At the 2 mg/kg Tookad^®^ dose, patients received increasing light doses of 230 and 360 J/cm^2^. Escalating the light dose showed an increasing volume of effectiveness, with all six patients responding at the highest light dose, resulting in lesions with up to 70% of the marginal zone affected. No critical adverse events were reported, and continence and efficacy were maintained. This trial demonstrated that Tookad^®^ VTP is safe and well-tolerated [74].

Subsequently, a phase II clinical study was conducted to investigate escalating light doses for the treatment of recurrent prostate cancer after the failure of external beam radiotherapy [74,75]. The study involved 28 patients who had previously participated in a phase I trial using Tookad^®^ 2 mg/kg vascular-targeted therapy. The patients received an intravenous injection of padoporfin, and 20 min later, 763 nm laser light was delivered to the prostate. The light dose was varied based on an evaluation of the prostate area treated during the previous phase I trial, with an average light dose of 32 J/cm^2^. Increasing the light dose enhanced the tissue response, with some patients showing up to 80% of the prostate affected, as determined by the 6-month biopsy. A complete response was observed when light doses of at least 23 J/cm^2^ were delivered in 90% of the prostate area, and 8 out of 13 patients who received this light dose were biopsy-negative for 6 months. Adverse effects were self-limited and modest in all patients except for two patients. The results indicated that Tookad^®^ VTP can produce large avascular regions in the irradiated prostate, leading to a complete negative-biopsy response at high light doses [75].

### 5.4. Padeliporfin

Padeliporfin (WST11, Tookad^®^ Soluble) has been approved in 31 countries of the EU, Mexico, and Israel to treat low-risk prostate cancer with VTP. On July 5, 2023, the FDA granted orphan drug designation to padeliporfin VTP for use as a potential therapeutic option in locally advanced pancreatic cancer patients. The water-soluble Tookad^®^ formulation was developed to prevent subclinical hepatotoxicity and cardiovascular events related to padoporfin [76].

A phase II clinical trial was conducted to evaluate medium-term cancer control and tolerability in patients with localized prostate cancer treated with VTP using the water-soluble Tookad^®^ (WST11). In this trial, 68 patients participated, and they received 4 mg/kg of WST11 with 200 J/cm^2^ light application. They were followed up for 3.5 years. At the end of the 3.5 years’ follow-up, an overall successful focal ablation was achieved in 51 patients (75%). Successful local ablation was accomplished in 51 patients (75%) after 3.5 years of follow-up. In the untreated area, cancer was observed in 17 patients (25%), and 34 patients (50%) showed no cancer in both prostate glands. There were 64 related side effects, and the majority of patients (95%) had adverse events below Clavien grade II [77].

### 5.5. Motexafin Lutetium

Motexafin lutetium, also known as Lutrin^®^ or Antrin^®^, has not been approved yet for the treatment of any cancer. A phase I trial of PDT with motexafin lutetium was performed in men who had locally recurrent prostate carcinoma to determine the maximally allowable dose. Fifteen patients with locally recurrent prostate cancer and no remote metastasis were involved, and 14 of them were treated. Motexafin lutetium was intravenously administered in the range of 0.5 to 2 mg/kg, 14 h, 6 h, or 3 h before exposure to 732 nm light at doses of 25–100 J/cm^2^. Light was delivered through trans-perineal brachytherapy via inserting optical fibers. All 14 patients completed the response on several 8 dose levels without dose-limiting toxicity, and no rectal or gastrointestinal toxicities were observed.

## 6. Breast Cancer

Breast cancer is the second most diagnosed cancer and the second leading cause of cancer mortality in women. According to breast cancer statistics 2022 from the American Cancer Society, about 13% of women will be diagnosed with invasive breast cancer, and 3% will die from breast cancer during their lifetime. The recurrence and metastasis of breast cancer remain major causes of death, despite recent technological advancements. Triple-negative breast carcinomas, in particular, have fewer therapy options and a high risk of recurrence and metastasis. Therefore, PDT has recently been studied as a potential treatment for breast cancer, offering a new and promising anti-carcinoma strategy that can be used alone or in combination with other approved or investigational treatment approaches across a wide range of applications.

Currently, there are no approved photosensitizers specifically for breast cancer treatment, but several preclinical and clinical trials are underway. Notably, Photofrin^®^, Foscan^®^, Laserphyrin^®^, Purlytin^®^, Verteporfin, and LuTex^®^ are being studied for breast cancer both preclinically and clinically.

### 6.1. Temoporfin

Temoporfin, also known as Foscan^®^ or mTHPC, has been approved for the treatment of head and neck cancer, but it has not yet been approved for the treatment of breast cancer. In a preclinical study, it was discovered that temoporfin and three-dimensional supramolecular-organic frameworks (SOFs) formed self-assembled nanoparticles that improved the effectiveness of oxygen (O_2_) generation. These SOFs and nanoparticles demonstrated biocompatibility and increased photocytotoxicity in four types of tumor cells, including human breast adenocarcinoma MCF7. The PDT mediated by temoporfin SOFs resulted in decreased IC_50_ values in MCF7 compared to temoporfin alone [78,79].

In another in vitro study, the induction of apoptosis and autophagy, cell uptake, effect on migration, and photodynamic activity of four novel synthesized non-symmetrical diarylporphyrins were compared with Foscan^®^ in human cancer cell lines, including breast adenocarcinoma cells (MCF7, MDA-MB231). The photodynamic activities of all four novel synthesized non-symmetrical diarylporphyrins in MCF7 and MDA-MB231 cells demonstrated cytotoxicity at sub-micromolar concentrations. The IC_50_ values were obtained following treatment with the synthesized non-symmetrical diarylporphyrins and Foscan^®^ for 24 h, 2 h of irradiation, and 24 h of incubation. The IC_50_ values of two synthesized non-symmetrical diarylporphyrins did not significantly differ from the IC50 value for Foscan^®^. However, all compounds inhibited cell migration, which is not an observed feature of Foscan^®^. Additionally, one of the four compounds indicated both anti-migratory effects and cytotoxicity in MDA-MB231 cells, which are representative of triple-negative breast cancer (TNBC), a type of cancer that is challenging to treat with current therapies such as endocrine therapy or targeted therapy [80].

Clinical trials have been conducted to demonstrate that mTHPC-mediated PDT provides minimally invasive treatment for recurrent chest wall breast cancer and has few side effects. In one study, three patients were given 0.1 mg/kg of mTHPC, and 48 h after administration, the light of 5 J/cm^2^ was illuminated. In another study, four patients were given 0.15 mg/kg of mTHPC, and 96 h after administration, the light of 10 J/cm^2^ was illuminated. As a result, PDT with mTHPC showed a complete response in all seven recurrent breast cancer patients [81].

### 6.2. Talaporfin Sodium

Talaporfin sodium is a Ce6 derivative, and its chemical structure is the tetra-sodium salt of mono-L-aspartylCe6 (NPe6) [20]. Talaporfin, also known as Laserphyrin^®^, is approved by the Ministry of Health, Labor and Welfare (MHLW), Japan for the treatment of lung and brain cancer, but has not yet been approved for breast cancer [62].

Both in vitro and in vivo studies have been conducted to demonstrate that cancer-targeting peptide p 18-4/Ce6-conjugated polyhedral oligomeric silsesquioxane (PPC) nanoparticles enhanced the ability of Ce6 to target TNBC cells, improving the efficacy of PDT. In an in vivo study, saline, free Ce6, or PPC was intravenously injected into MDA-MB-231 tumor-bearing mice. After 24 h, a 670 nm laser was delivered to the tumor site for 12 min. Among them, the mice injected with PPC showed a significantly reduced tumor growth. Additionally, PPC induced ROS production and substantial apoptosis in cancer cells [82].

Another in vitro study evaluated the efficacy of bifunctional theranostic nanoprobes (BN) during PDT in TNBC cells. BN is a gold nanoparticle conjugated with Ce6 and epidermal growth factor (EGF). Ce6 is stabilized when attached to the gold nanoparticles. BN was applied at various concentrations (0 μg/mL to 1.20 μg/mL) in each well, and then 660 nm light was delivered. A gradual absorption of nanoprobes in TNBC cells was observed, and high cytotoxicity to TNBC cells was also observed at 0.2 μg/mL BN concentration. At this concentration, 58% of cancer cells underwent necrosis, and 38% underwent apoptosis, while ROS levels were 9 times higher in cancer cells than in normal cells. This study indicated that PDT with EGF-Ce6-gold nanoparticles increased ROS levels, leading to efficient apoptosis in TNBC cells, without affecting normal cells [83].

A phase I clinical trial of cutaneous disease, including breast cancer, was carried out using NPe6 with PDT. Eleven patients with a total of 14 cancer sites were treated with PDT using NPe6. Four hours after the injection of NPe6, the tumor was exposed to light with a wavelength of 664 nm. The dosage of NPe6 was gradually increased from 2.5 to 3.5 mg/kg. There were no side effects from the intravenous injection of NPe6 at the maximum 3.5 mg/kg dose, with the exception of common photosensitivity. In all patients, the tissue was blanched immediately by light treatment, followed by a necrosis of the tumor. Regression of the tumor occurred over 24–48 h after the light therapy, and a thick eschar was formed in the tumor area [84].

Laserphyrin^®^ can also be used for lymph node biopsy in breast cancer patients. Sentinel lymph node biopsy using Laserphyrin^®^ in breast cancer patients is considered to be useful as a complementary method to other current methods. In 14 of 21 patients, the Laserphyrin^®^ method successfully detected sentinel nodes [85]. In these clinical trials, no side effects, including photosensitivity, were encountered. Therefore, sentinel node navigation biopsy (SNNB) using Laserphyrin^®^ is considered to be practicable [86].

### 6.3. Aluminum Phthalocyanine

The effect of PDT-mediated hydroxyl aluminum phthalocyanine (AlOH-PC) was tested for mammary carcinoma compared to Metvix^®^-PDT. MDA-MB-231 cells, which are human mammary adenocarcinoma cell lines, were injected into athymic nude mice. After topical application of the photosensitizer, the irradiation of light was conducted at 600–700 nm. This study showed that AlOH-PC treatment resulted in a complete reduction in tumors in 90% of test animals, while the use of Metvix^®^ only delayed tumor growth [87].

Photosense received regulatory approvals from 2001 to 2008 in Russia. It is a water-soluble mixture of sulfonated aluminum phthalocyanines (AlPcS) and has been studied for the treatment of BCC, lung cancer, head and neck cancer, gastric cancer, and breast cancer metastases in Russia [20].

A phase III clinical trial showed that PDT with photosense is effective in treating breast cancer as a pre-operative treatment and as palliation in cases of recurrence. A total of 24 patients with breast cancer were treated with photosense at 0.5 mg/kg, and then light was irradiated at 150–200 J/cm^2^ after 2–4 h later. Overall, this trial demonstrated the noticeable effectiveness and feasibility of preoperative PDT mediated by photosense for breast cancer treatment.

### 6.4. Verteporfin

Verteporfin, also known as Visudyne^®^, was approved by the FDA in 2000 for the treatment of choroidal neovascularization [20], but it has not been approved for the treatment of breast cancer yet. A phase I/IIa dose escalation study was performed in 12 female patients diagnosed with invasive ductal breast cancer and planned to undergo mastectomy as a first therapy. Intravenous administration of 0.4 mg/kg verteporfin was followed by exposure to increasing light doses (20, 30, 40, 50 J/cm^2^). MRI scans were performed before and after PDT, and a histological examination of the excised tissue was also conducted. PDT effects were observed via MRI in seven patients and through histology in eight patients, with the effects increasing in scale with the delivered light dose, showing a good correlation between the two groups. Histologically distinctive features of PDT necrosis were identified in contrast to spontaneous necrosis. This study established the potential efficacy of PDT in the treatment of early breast cancer [88].

### 6.5. Porfimer Sodium

In 1993, Photofrin^®^ was first approved in Canada as a treatment for bladder cancer [20] and it has since been approved in Japan and by the US FDA for other cancers. However, it has not been approved for the treatment of breast cancer.

In a clinical trial, patients with chest wall progression of breast cancer were treated with low-dose Photofrin^®^-mediated PDT, resulting in a significant clinical response. Fourteen patients with over 500 chest wall metastases were enrolled in this trial. All patients received an intravenous injection of 0.8 mg/kg Photofrin^®^, followed by light irradiation at 150 to 200 J/cm^2^. The follow-up period ranged from 6 to 24 months. Tumor necrosis was observed in all patients, and 9 out of 14 patients showed a complete response, even for lesions with a thickness of 2 cm. Another phase I clinical trial was conducted to establish the maximum tolerated dose (MTD) of continuous low-irradiance PDT (CLIPT) using Photofrin^®^ and 630 nm light. Nine patients with chest wall progression of breast cancer, who had previously failed surgery and radiation therapy, participated in this trial. All patients received intravenous Photofrin^®^ at 0.8 mg/kg before starting CLIPT. The initial two patients treated with light at 100 J/cm^2^ exhibited incomplete thickness skin necrosis, leading to a reduction in light dose for the subsequent patients to 50 J/cm^2^. The following seven patients did not experience partial or full thickness necrosis, and the MTD was determined to be 50 J/cm^2^ over 24 h. Six out of nine patients (67%) showed a complete or partial clinical response, and two patients had a considerable degeneration of tumor nodules distant from the treatment field [89].

### 6.6. SnET2

Tin ethyl etiopurpurin (SnET2) is also known as Purlytin^®^, rostaporfin or photrex [20]. It is currently undergoing clinical trials for the treatment of breast, bile duct, ovarian, and colon cancer [62]. A phase II/III clinical trial was conducted to evaluate the efficacy of Purlytin^®^-mediated PDT in recurrent cutaneous metastatic breast cancer. Eight patients with chest wall recurrence of cutaneous breast cancer, despite prior surgery, chemotherapy, and radiotherapy, were treated in this trial. Each patient received an intravenous injection of 1.2 mg/kg of Purlytin^®^, followed by PDT 24 h later using laser light at 657–663 nm. The 6-month follow-up showed response rates of 92% for complete response, 8% for partial response, and 0% for no response after photodynamic therapy. The treatment was well-tolerated, with minimal morbidity and no systemic toxicity reported. Additionally, there were no adverse reactions of photosensitivity observed in these patients [90].

### 6.7. Motexafin Lutetium

Motexafin lutetium is also known as lutetium texaphyrin, LuTex^®^, Lutrin^®^ or Antrin^®^ [20]. A phase II clinical trial was conducted for locally recurrent breast cancer using LuTex^®^-mediated PDT. The trial included women who had previously failed radiotherapy for large tumors. Twenty-five patients were enrolled and received two consecutive treatments, 21 days apart, with an escalation of the LuTex^®^ dose to 1.5 and 3.5 mg/kg. The light treatment at a wavelength of 732 nm was delivered 4 h after the injection of LuTex. Except for one patient, all others achieved a complete response with LuTex^®^-mediated PDT treatments [91]. LuTex^®^ is water-soluble and is known to exhibit rare mild photosensitivity, making it a significant advantage for clinical application [81].

### 6.8. Meso-5-[p-diethylene triamine pentaacetic acid-aminophenyl]-10,15,20-triphenyl-porphyrin

In vitro, MCF7 (human breast adenocarcinoma) and MCF7/ADR cells (adriamycin-resistant, P-gp overexpressed cells) were incubated with meso-5-[p-diethylene triamine pentaacetic acid-aminophenyl]-10,15,20-triphenyl-porphyrin (DTP) at concentrations ranging from 0.20 to 6.25 μM for 24 h, followed by light irradiation at 650 nm for 10 min. The results demonstrated that PDT mediated by DTP significantly reduced the expression of the MDR1 gene in the MCF7 cell line, indicating its potential as a photosensitizer for treating multidrug-resistant breast cancer in humans [92].

### 6.9. Zinc Phthalocyanine

An in vitro study was conducted to investigate the cell death pathway in breast cancer cell lines (MCF7) using zinc phthalocyanine (ZnPcS_mix_)-mediated PDT with 680 nm light at an intensity of 5 J/cm^2^. After treatment, an increased number of apoptotic cells were observed. The expression of genes related to apoptosis, including B-cell lymphoma 2 (Bcl-2), DNA fragmentation factor alpha (DFFA), and caspase 2 (CASP2), was upregulated, indicating ZnPcS_mix_-PDT induced apoptotic cell death pathway and stimulated cell death initiation [93].

**Table 1:** Photosensitizers used in basic research for cancer treatment.

Cancer Type	Photosensitizer	Wavelength (nm)	In Vitro/ In Vivo	Outcome (Year)
Lung Cancer	Ce6	650	in vitro	Promoted systemic immune responses via sequential PDT and PTT using dual-modal single-walled carbon nanohorns (2020) [26]
	AlPcS_4_Cl	673.2	in vitro	Effective in treating lung cancer (2019) [33]
		673.2	in vitro	Enhanced drug delivery of AlPcS_4_Cl-gold nanoparticle–antibody conjugates in lung cancer stem cells (2020) [32]
		673.2	in vitro	Induced apoptosis and necrosis in lung cancer stem cells (2021) [34]
	Verteporfin	689 ± 3	in vivo (mouse)	Improved efficacy of Visudyne^®^-PDT with Perftoran^®^ via suppressing hypoxia pathway in murine lung cancer (2022) [18]
Head and Neck Cancer	Temoporfin	670	in vitro	Boosted cancer cell killing and antitumor efficacy combined with fenretinide (2016) [42]
	Redaporfin	748	in vivo (mouse)	Induced immune responses via redaporfin-vascular-PDT in mice with tumors (2020) [46]
	IRDye700DX	690	in vitro	Induced selectivity and tumor cell death through EGFR-targeted nanobody–photosensitizer conjugates (2016) [48]
Non-Melanoma Skin Cancer	Methyl 5-aminolevulinate	630	in vitro	Improved cytotoxic effect on PDT-resistant skin cancer squamous cells with epigallocatechin gallate (2020) [55]
	5-Aminolevulinic acid	621	in vitro	Improved anti-tumor efficacy with gold nanoparticles conjugated to 5-ALA (2020) [60]
Prostate Cancer	Temoporfin	652	in vitro	Improved intracellular uptake and enhanced photodynamic activity using PEGylated liposomal formulation of mTHPC (2012) [66]
	5-Aminolevulinic acid	629	in vitro	Overcame ALA-PDT resistance with ABCG2 transporter inhibition by fumitremorgin C (FTC) (2021) [69]
	Padoporfin	650–800	in vivo (mouse)	Effective in treating local and disseminated small-cell carcinoma of the prostate (SCCP) [73]
Breast Cancer	Temoporfin	-	in vivo (mouse)	Effective in suppressing tumor growth with SOFs-temoporfin self-assembled nanoparticles (2022) [78]
		580	in vitro	More effective in inducing cell death with positively charged diaryl porphyrins compared with Foscan^®^ (2019) [80]
	Talaporfin sodium	670	in vitro	Enhanced retention in tumor tissues, effectively targeted and inhibited the growth of breast cancer with PPC nanoparticles (2020) [82]
		660	in vitro	Effective in TNBC with EGF-Ce6 functionalized gold nanoparticles (2021) [83]
	Aluminium phthalocyanine	600–700	in vitro	Effective in complete mammarian carcinoma remission with AlOH-PC (2012) [87]
	DTP	650	in vitro	Effective in MDR1 over-expressing breast cancer cells via DTP-PDT with adriamycin (2017) [92]
	Zinc phtalycyanine	680	in vitro	Enhanced apoptotic cell death pathway and stimulated programmed cell death with ZnPcS_mix_-PDT (2014) [93]

**Table 2:** Photosensitizers used in ongoing clinical trials for cancer treatment.

Cancer Type	Photosensitizer	Phase	Estimated Enrollment	Study Title	ClinicalTrials.gov Identifier
Lung Cancer	Porfimer sodium	I	12	Light Dosimetry for Intraoperative Photodynamic Therapy (IO-PDT) With Porfimer Sodium (Photofrin) in Patients With Malignant Mesothelioma or Non-Small Cell Lung Cancer (NSCLC), or Other Malignancies With Pleural Disease—Phase I Study	NCT03678350
		I/II	65	Endobronchial Ultrasound Transbronchial Needle Guided Interstitial Photodynamic Therapy for Palliation of Locally Advanced Lung Cancer and Advanced Cancers Obstructing the Airway—Phase I/II Lung Cancer	NCT03735095
		I	16	Utilizing Photodynamic Therapy to Amplify the Response to Immunotherapy in Patients With Non-Small Cell Lung Cancer (NSCLC) With Pleural Disease or Malignant Pleural Mesothelioma (MPM)—Phase I Study	NCT04836429
Head and Neck Cancer	ASP-1929	III	275	A Phase 3, Randomized, Double-Arm, Open-Label, Controlled Trial of ASP-1929 Photoimmunotherapy Versus Physician’s Choice Standard of Care for the Treatment of Locoregional, Recurrent Head and Neck Squamous Cell Carcinoma in Patients Who Have Failed or Progressed On or After at Least Two Lines of Therapy, of Which at Least One Line Must Be Systemic Therapy	NCT03769506
	Porfimer sodium	I/II	82	A Randomized, Phase 2 Trial With a Phase 1 Safety Run-in: Porfimer Sodium Mediated Interstitial Photodynamic Therapy and Standard of Care (SoC) Therapy Versus SoC Therapy Alone for the Treatment of Patients With Locally Advanced or Recurrent Head and Neck Cancer	NCT03727061
	5-Aminolevulinic acid hydrochloride	II	26	The Role of 5-Aminolevulinic Acid Fluorescence-Guided Surgery in Head and Neck Cancers: A Pilot Trial	NCT05101798
Non-Melanoma Skin Cancer	5-Aminolevulinic acid	II	28	Alteration of the Immune Microenvironment in Basal Cell Carcinoma (BCC) Following Photodynamic Therapy (PDT)	NCT05020912
		III	186	A Randomized, Double Blind, Vehicle-controlled Multicenter Phase III Study to Evaluate the Safety and Efficacy of BF-200 ALA (Ameluz^®^) and BF-RhodoLED^®^ in the Treatment of Superficial Basal Cell Carcinoma (sBCC) With Photodynamic Therapy (PDT)	NCT03573401
Prostate Cancer	Tookad^®^ Soluble	II	50	Study of the Efficacy, Safety and Quality of Life After TOOKAD^®^ Soluble Vascular Targeted Photodynamic Therapy (VTP) for Minimally Invasive Treatment of Localized Intermediate Risk Prostate Cancer	NCT03315754
	Verteporfin	I/II	66	Open-label Clinical Study to Assess the Safety and Efficacy of the SpectraCure P18 System (Interstitial Multiple Diode Lasers and IDOSE^®^ Software) and Verteporfin for Injection (VFI) for the Treatment of Recurrent Prostate Cancer	NCT03067051

## 7. Conclusions

PDT has emerged as a promising and evolving treatment modality for various cancers. This review highlights the recent trends in PDT research and clinical trials across different cancer types, including lung cancer, head and neck cancer, non-melanoma skin cancer, prostate cancer, and breast cancer. The use of various photosensitizers in PDT has shown encouraging results in both basic research and clinical settings. PDT’s potential as a less invasive and targeted therapy, combined with ongoing efforts to optimize treatment protocols and enhance photosensitizer delivery, holds great promise for advancing cancer treatment and improving patient outcomes. It should be noted that there also exist some limitations of PDT. PDT may lead to unwanted adverse effects in some cases and light delivery is limited to specific location and size of tumors. Further research and clinical trials are essential to harness the full potential of PDT in personalized cancer management. Combination strategies of PDT with other conventional cancer treatments are actively being developed.

## Data Availability

Data sharing not applicable.

## Abbreviations

PDT	photodynamic therapy
ROS	reactive oxygen species
CSC	cancer stem cell
FDA	Food and Drug Administration
Ce6	chlorin e6
PTT	photothermal therapy
DAMP	damage-associated molecular pattern
COF	composite-type optical fiberscope
CELC	central early stage lung cancer
AuNP	gold nanoparticle
Ab	antibody
VEGF	vascular endothelial growth factor
HPPH	2-[1-hexyloxyethyl]-2-devinyl pyropheophorbide-a
CIS	carcinomas in situ
MIC	microinvasive cancer
SCC	squamous cell carcinoma
mTHPC	m-tetrahydroxyphenylchlorin
EMA	European Medicines Agency
HPR	N-(4-hydroxyphenyl) retinamide
QOL	quality of life
EGFR	epidermal growth factor receptor
TPCS2a	disulfonated tetraphenyl chlorine
BCC	basal-cell carcinoma
5-ALA	5-aminolevulinic acid
AK	actinic keratosis
MAL	methyl aminolevulinate
EGCG	epigallocatechin gallate
MHRF	Ministry of Health of the Russian Federation
PpIX	protoporphyrin IX
ABCG2	ATP-binding cassette transporter subfamily G2
PSA	prostate-specific antigen
VTP	vascular-targeted PDT
SOF	supramolecular organic framework
MHLW	Ministry of Health, Labor and Welfare
TNBC	triple-negative breast cancer
BN	bifunctional theranostic nanoprobe
SNNB	sentinel node navigation biopsy
MTD	maximum tolerated dose
CLIPT	continuous low-irradiance PDT
Bcl-2	B-cell lymphoma 2
DFFA	DNA fragmentation factor alpha
CASP2	caspase 2
